# Association of Motor Stereotypies with Emotional Dysregulation and Anxiety/Depression Symptoms in Children with Autism Spectrum Disorder

**DOI:** 10.3390/children13101363

**Published:** 2026-10-08

**Authors:** María Gema Hurtado Ruíz, María Jesús Arranz Calderón, Víctor Pérez Solá, Mariona Prades Oropesa, Amaia Hervás Zúñiga

**Affiliations:** 1 Hospital del Mar Research Institute, 08003 Barcelona, Spain; vperezsola@hmar.cat; 2 Research Laboratory Unit, Fundació Docència i Recerca Mútua de Terrassa, 08221 Terrassa, Spain; mjarranz@mutuaterrassa.es; 3 Department of Health and Life Sciences, Universitat Pompeu Fabra, 08003 Barcelona, Spain; 4 Biomedical Investigation Centre Network of Mental Health (CIBERSAM), 28029 Madrid, Spain; 5 Fundació Recerca i Docència Mútua de Terrassa, 08221 Terrassa, Spain; mprades.cm@gmail.com; 6 Child and Youth Mental Health Service, Hospital Universitari Mútua de Terrassa, 08221 Terrassa, Spain; ahervas@mutuaterrassa.es

**Keywords:** motor stereotypies, emotional dysregulation, restricted and repetitive behaviours, anxiety, depression, autism spectrum disorder

## Abstract

**Highlights:**

**What are the main findings?**

- Frequent motor stereotypies were significantly associated with emotional dysregulation and with anxiety/depression symptoms in children with ASD.
- No significant association was found between other restricted and repetitive behaviours and emotional dysregulation.

**What are the implication of the main finding?**

- Evaluating the frequency of motor stereotypies could help identify emotional dysregulation in children with ASD.
- These findings underscore the clinical importance of targeting emotional dysregulation in therapeutic interventions with children with ASD and frequent motor stereotypies.

**Abstract:**

**Background:** Comorbidities such as anxiety and challenging behaviours are common in children with autism spectrum disorder (ASD). These comorbidities are often linked to emotional dysregulation (ED) and are frequently accompanied by motor stereotypies (MS). **Objective**: This study aimed primarily to examine the association between lifetime motor stereotypies (LMS) and both emotional dysregulation (ED) and anxiety/depression symptoms (ADS) in children with ASD. Additionally, a secondary objective was to explore the association between ED and the lifetime broader spectrum of restricted and repetitive behaviours (RRBs), and their specific subtypes. **Methods:** The study included 151 children with ASD (ages 3–11). ADS and ED were assessed using the Child Behaviour Checklist (CBCL) and the Child Behaviour Dysregulation Profile (CBCL-DP), respectively, while LMS and lifetime RRBs were evaluated via the Autism Diagnostic Interview-Revised (ADI-R). Data were analyzed using regression models. **Results:** Frequent LMS were significantly associated with ED and ADS. Gender, intellectual disability (ID), and ASD severity did not significantly influence these relationships. Age had a significant effect on the association between frequent LMS and ADS. No significant correspondence was found between ED and lifetime RRBs in general or with subtypes of RRBs. **Conclusions**: Our findings suggest that frequent LMS are associated with ED and ADS in children with ASD. Identifying ASD subgroups based on the presence of LMS may contribute to the development of detection methods and targeted and effective interventions for ED. Differences observed between LMS and other RRBs in relation to ED support the notion that low- and high-level repetitive behaviours involve distinct clinical features and should be studied independently.

## 1. Introduction

Autism spectrum disorder (ASD) is a neurodevelopmental condition characterized by deficits in social communication and interaction, as well as restricted and repetitive patterns of behaviour, interests, or activities. ASD symptoms appear in early childhood and affect daily functioning [1].

Restricted and repetitive behaviours (RRBs) in ASD refer to actions that are topographically similar across contexts, conducted in unsuited situations, and are performed repetitively [2]. Bishop et al. (2013) proposed a classification of these behaviours into lower- and higher-order categories. Lower-order behaviours include sensorimotor actions such as motor stereotypies (MS), repetitive object use, and unusual sensory interests [3]. Higher-order behaviours involve cognitive rigidity, such as insistence on sameness, rituals, and resistance to change [3]. This classification is supported by factor analyses using the Autism Diagnostic Interview-Revised (ADI-R) and is associated with distinct clinical features [4,5,6], neuroanatomical correlates [7,8,9], and genetic bases [4,6,10].

MS are defined as fixed, involuntary, rhythmic, and purposeless movements with repetitive patterns [11]. While they can occur in typically developing children under the age of four and in subjects with a variety of disorders [12,13,14], they are more prevalent (from 22% to 98% of cases), and display more variation in topography and severity, in individuals with ASD [15,16,17].

RRBs in ASD can serve adaptive functions, such as seeking sensory input or arousal, gaining attention or access to objects, or avoiding unwanted situations, when there is a lack of cognitive abilities that are still to be developed, managing the environment by increasing predictability, reducing anxiety, and improving concentration and emotional regulation [18,19,20,21,22,23,24,25].

Up to 82% of individuals with ASD exhibit emotional dysregulation (ED), defined as deficits in cognitive, physiological, and behavioural processes that modulate emotional responses. ED is related to the core symptoms and severity of ASD, although with some mixed results [25,26,27,28,29]. Other associated features of ASD also contribute to ED, including difficulties with sleep and feeding, speaking ability, shifting attention appropriately, understanding and expressing emotions, executive functions, parents’ well-being, among others [28,30,31]. As a result, individuals with ASD may exhibit increased negative affect (such as anxiety and anger), reduced positive affect, and use maladaptive strategies or have difficulties in regulating positive emotions; however, other studies have found no support for these findings [32,33,34,35]. Without effective ER, individuals with ASD experience longer and more severe periods of distress, which may predispose them to the development of depression and anxiety [34]. The link between anxiety or depression and ED has been described in several publications [27,32,36,37,38,39,40].

Anxiety in ASD increases with the severity of autism [17] and may stem from social communication difficulties, sensory processing issues, physiological and neurological processes related to impaired cognitive control, regulatory processes, and arousal, intolerance of uncertainty and changes in routine, among others [19,23,24,38].

Several publications describe the relationship between ED and RRBs [21,25,26,32,41,42,43,44], between anxiety and RRBs [20,21,40,43,44,45] and between anxiety and some specific high-level RRBs [20,24,25,43,44,45].

Few studies have studied the association of ED with repetitive sensorimotor behaviours (RSMB) showing a positive relation [25,42,46,47], while other research [48] has yielded conflicting results. Conversely, numerous studies have documented associations between anxiety and MS [19,20,21,24,25,49] although some results are also contradictory [43,44].

The association between MS and ED or anxiety/depression symptoms (ADS) may be regulated by clinical and demographic factors. Younger, non-verbal children with intellectual disability and more severe autistic symptoms exhibit greater frequency and intensity of MS and higher levels of hyperactivity and irritability [17,21,29,47,50,51,52,53,54]. With age, ASD children become less dysregulated [42] and some repetitive stereotyped behaviours can be substituted by repetitive ruminative thoughts of high emotional content, disruptive behaviours, major affective disorders, and anxiety, reflecting a diachronic shift from overt motor manifestations toward more cognitively mediated or internalizing components of emotional dysregulation [26,29,52], though persistent severe dysregulation remains evident in specific subgroups [28,31,55].

This study investigates the association between lifetime MS (LMS) and ED and ADS in children under 12 years of age with ASD. We hypothesize that children with frequent LMS will exhibit greater ED and ADS. Furthermore, we examine whether other types of lifetime RRBs are also linked to ED. Identifying such associations could contribute to the detection of young children with ED through the presence of frequent LMS and to understanding the function of MS and thus lead to the development of targeted interventions for children with ASD exhibiting frequent LMS and ED.

## 2. Materials and Methods

A cross-sectional observational study with retrospective data was conducted to describe the association between lifetime RRBs and ED and between LMS and ED and anxiety/depression symptoms in children with ASD who are under 12 years of age.

### 2.1. Sample and Procedures

A total of 151 children with ASD, all under the age of 12 (mean age = 7.14 [SD 0.17] years, range 3–11 years; 83.5% male), were consecutively recruited from a Child and Adolescent Psychiatry Service between 2003 and 2017. All participants had a confirmed diagnosis of ASD based on DSM-IV-TR criteria [56] and the ADI-R (WPS, Los Angeles, CA, USA) [57]. Children with severe cranial trauma or significant sensory impairments (which could manifest symptoms similar to those of ASD) were excluded from the study to ensure a more homogeneous primary ASD clinical sample, along with those with incomplete clinical assessment data or a negative diagnostic outcome on the ADI-R. Assessments— including the standardized evaluation instruments—were administered concurrently at the baseline evaluation. ID was identified from clinical records using DSM-IV-TR criteria and IQ ≤ 70 assessed via standardized instruments tailored to the participant’s developmental and linguistic profile: Wechsler Intelligence Scale for Children (WISC-IV and WISC-III; PsychCorp, San Antonio, TX, USA) [58,59] for older children; the McCarthy Scales of Children’s Abilities (The Psychological Corporation, New York, NY, USA) [60] for younger developmental stages; and the Kaufman Assessment Battery for Children (K-ABC; Pearson, Bloomington, MN, USA) [61] for children requiring alternative cognitive formatting. Of the participants, 18.8% had ID, 39.7% had language delay, and 71% exhibited fluent speech (language assessed by ADI-R and structured clinical interviews) (Table 1).

### 2.2. Measures

#### 2.2.1. Autism Diagnostic Interview-Revised (ADI-R)

The ADI-R Spanish version [57] is a semi-structured interview of 93 questions addressed to parents or caregivers, with interviewer-based scoring, the results of which are organized into four domains that explore language/communication, reciprocal social interactions, restricted, repetitive and stereotyped behaviours and interests and development. The diagnostic algorithm is used to establish cut-off points for autism and autism spectrum disorder.

Restricted and Repetitive Behaviours. RRBs were assessed via Domain C of the ADI-R algorithm (restricted, repetitive, and stereotyped patterns of behaviour). The punctuation of the RRB domain of ADI-R (C), and their subdomains were assessed: circumscribed interests (C1), compulsions/rituals (C2), stereotyped and repetitive motor mannerisms (C3), and preoccupations with parts of objects (C4). RSMB were calculated by summing the scores for the following ADI-R items: unusual preoccupations; repetitive use of objects; hand and finger mannerisms; other complex mannerisms; and unusual sensory interests [62]. MS were assessed using the ADI-R C3 diagnostic algorithm score (stereotyped and repetitive motor mannerisms), obtained by selecting the highest value between Item 77 (‘Hand and finger mannerisms’) and Item 78 (‘Other complex mannerisms’) of the interview. Children scoring ≥2 were classified as having frequent lifetime MS (LMS), while those scoring 0–1 were classified as having absent or occasional LMS. This threshold distinguishes clear and frequent abnormality (score of 2) from possible abnormality (score of 1) to prevent any confounding effects stemming from borderline phenotypes.

ASD Severity. ASD severity was evaluated using Domain A (reciprocal social interaction) and non-verbal items from Domain B (communication) of the ADI-R [63]. Only the non-verbal subdomains (B1 + B4) from ADI-R were employed to include the full cohort in the analysis. The ADI-R scores of domains A and B (non-verbal) were aggregated to form a composite index: social reciprocity and non-verbal communication index [64,65].

#### 2.2.2. Child Behaviour Checklist (CBCL 4–18)

The Spanish version of the CBCL 4–18 (ASEBA, Burlington, VT, USA) [66], a parent-report measure with 113 items, was utilized. It includes eight syndrome subscales (Anxious/depressed, Withdrawn/depressed, Somatic complaints, Social problems, Thought problems, Attention problems, Rule-breaking behaviour, and Aggressive behaviour) and three composite dimensions: internalizing; externalizing; and total problems. There are cut-off points for considering clinically significant behaviour in each of them.

Emotional Dysregulation. ED was defined using the CBCL-Dysregulation Profile (CBCL-DP) cut-off score ≥ 180, calculated as the sum of the anxiety/depression, aggression, and inattention subscale T-scores. It corresponds to deficient emotional self-regulation (DESR), considered as moderate ED. Severe emotional dysregulation (CBCL-DP cut-off score ≥ 210) was included solely for descriptive sample characterization, as this was not a target of our association analyses [26,67]. The CBCL-DP has been specifically validated for ASD [30,42,68].

Anxiety/Depression Symptoms. Anxiety/depression symptoms were assessed using the CBCL anxiety/depression subscale. The cut-off point for clinically significant behaviour was a T-score ≥ 70.

All assessments were conducted by trained psychologists and psychiatrists with expertise in administering the ADI-R.

### 2.3. Statistical Analyses

Descriptive statistics were calculated, reporting frequencies and percentages for categorical variables, and medians with interquartile ranges (IQRs) for non-normally distributed continuous data. Normality was assessed using the Kolmogorov–Smirnov test with Lilliefors correction.

To examine the association between ED and RRBs and RRB subtypes, univariate logistic regression models were employed. Multivariable logistic regression models were constructed to evaluate the association between LMS (independent variable) and both ED and ADS (dependent variables). So, LMS was entered into two separate models; each adjusted for age and social reciprocity and non-verbal communication index (as this covariate has been identified as clinically relevant in the previous literature). Missing data in the regression models were handled via listwise deletion, ensuring that analyses were conducted on complete cases for each model. Potential multicollinearity among independent variables was assessed and ruled out using Variance Inflation Factors (VIF). The definitive selection of covariates was guided jointly by existing literature and established clinical relevance, combined with preliminary statistical analyses and univariate logistic regression of possible covariates for the LMS and ED association to ensure a robust and well-grounded multivariable model. Preliminary analyses showed that age was associated only with ED and ADS, while social reciprocity and non-verbal communication index was associated only with LMS and fluent speech was associated only with ADS (*p* < 0.05). Gender and intellectual disability were not related to either outcome (*p* > 0.05) (Table S1). Fluent speech was excluded from the regression model due to the strong correlation between verbal and non-verbal communication observed in our sample (rs = 0.801 *p* < 0.001) pointing to a risk of collinearity within these closely related clinical domains [69,70].

Statistical analyses were conducted using IBM SPSS Statistics version 22 (IBM, Armonk, NY, USA).

## 3. Results

### 3.1. Clinical Characteristics

LMS, as measured by item C3 of ADI-R, were classified as frequent in 32.5% of the children and 67.6% as occasional or absent LMS (occasional in 23.2%, and absent in 44.4%). The median score for stereotyped and repetitive motor mannerisms (C3) was one (IQR 2). ED was present in 57% of the cohort. Furthermore, 14.6% of the sample exhibited severe ED. Analysis of the CBCL subscales that form part of the CBCL-DP indicated that 17.2% of the children scored in the clinically significant range for anxiety/depression, 39.1% for inattention, and 10.6% for aggressive behaviour (Table 1). As expected, a significant positive correlation was observed between CBCL Dysregulation Profile (CBCL-DP) and the CBCL Anxiety/Depression subscale scores in our sample (r_s_ = 0.736, *p* < 0.001), reflecting the fact that anxiety and depression symptoms represent core constituents embedded within the broader dysregulation profile.

### 3.2. Restricted and Repetitive Behaviours and Emotional Dysregulation

Univariate logistic regression analyses were conducted to investigate the association between global RRBs and subtypes of RRBs with ED. The analyses revealed no significant associations between ED and global RRBs (ADI-R Domain C, OR 1.036, *p* = 0.631) or other specific subtypes, including circumscribed interests (C1), compulsions/rituals (C2), preoccupations with part-objects (C4), or RSMB. Stereotyped and repetitive motor mannerisms (C3) showed a marginal association with ED in this analysis (OR 1.436, *p* = 0.063) (Table 2).

### 3.3. Motor Stereotypies and Emotional Dysregulation

In the univariate regression analysis, LMS were significantly associated with ED (OR 2.268, 95% CI [1.089, 4.725], *p* = 0.029). Age (OR 1.155, *p* = 0.074) was marginally associated with ED, while sex, ID, social reciprocity and non-verbal communication index was not significant variables in this analysis (Table 3).

The multivariable logistic regression model examining the association between LMS and ED, adjusting for age and social reciprocity and non-verbal communication index, was statistically significant (χ^2^(3) = 9.383, *p* = 0.025). Of the 151 total cases, 150 were included in the final regression model, with only one case (0.7%) excluded due to missing data. Frequent LMS were significantly associated with higher odds of ED (OR 2.576, 95% CI [1.184, 5.606], *p* = 0.017). Age showed a marginal association (OR 1.174, *p* = 0.055). Social reciprocity and non-verbal communication index was not a significant covariate in the model (*p* = 0.435) (Table 4).

### 3.4. Motor Stereotypies and Anxiety/Depression Symptoms

In the univariate regression analysis, LMS (OR 2.676, *p* = 0.025), age (OR 1.430, *p* = 0.001), and fluent speech (OR 5.441, *p* = 0.025) were significantly associated with ADS (Table 5).

The multivariable logistic regression model examining the association between LMS and ADS, adjusting for age and social reciprocity and non-verbal communication index, was statistically significant (χ^2^(3) = 19.412, *p*= 0.001). Of the 151 total cases, 150 were included in the final regression model, with only one case (0.7%) excluded due to missing data. Frequent LMS were significantly associated with higher odds of clinically significant ADS (adjusted OR 3.310, 95% CI [1.266, 8.654], *p* = 0.015). Age also remained a highly significant predictor (adjusted OR 1.520, *p* = 0.001). Social reciprocity and non-verbal communication index was not a significant covariate in this model (*p* = 0.431) (Table 6).

## 4. Discussion

This study investigated the relationships between LMS and ED and LMS and ADS in a cohort of children with ASD. Our findings demonstrate a significant association between frequent LMS and both ED and ADS, with age influencing the latter relationship. However, given the relatively small number of participants exhibiting clinically significant ADS, these specific estimates should be treated as exploratory and interpreted with appropriate caution. Interestingly, our results demonstrate a lack of association between ED and global lifetime RRBs or other specific lifetime RRB subtypes.

The prevalence of frequent LMS (32.4%), ED (57%), and ADS (17.2%) in our sample aligns with established literature [17,30,39,71].

While some studies have linked ED to global or high-level RRBs [25,42,46,47,72] our lack of such an association in this age group is consistent with findings by Conner et al. (2020) [40]. Furthermore, we did not find the previously described link between ED and RSMB [25], and the observed association between LMS and ED was only marginally significant, suggesting a need for further research. These discrepancies across studies may stem from the specific measures used for MS and ED and from differences in age ranges [30,31,42,73,74]. Different ED trajectories over time have been described depending on autistic traits, some clinical characteristics and family issues [28,31,75,76]. RRBs also change depending on the developmental stage: RSMB typically predominate in children under the age of four, while low-order and high-order RRBs emerge in childhood and later childhood, respectively [73,77]. Compared to other RRBs, the distinct relationship observed between LMS and ED, although marginally significant, supports the theory that low- and high-order repetitive behaviours involve different clinical features and potentially distinct neuroanatomical and genetic correlates [4,6,9,46,72,78,79,80,81,82] and should be investigated independently [12,36,72,83].

Consistent with research linking RSMB to ED, our multivariable logistic regression results show that children under 12 with frequent LMS are significantly more likely to experience ED [25,26,42,46], but contrasts with some recent studies [47,48]. On the other hand, our finding of a link between LMS and ADS aligns with most of the existing literature [19,20,21,24,25,41,49] albeit with some discrepancies [43,44]. Notably, these findings hint at the possibility that a lifetime history of frequent MS may act as an indicator to help detect current ED and ADS. Dedicated longitudinal studies are warranted to rigorously test this hypothesis. Beyond this interpretation, however, several alternative explanations for this association must also be considered.

Neurobiologically, the link between MS and emotional manifestations may involve shared genetic factors [84], neurotransmitter imbalances (GABA-glutamate and serotonin) [78] and dysfunction in brain circuits, such as the limbic system, the hypothalamic–pituitary–adrenal axis, and the cortical–basal ganglia–thalamic circuitry [22,79,82,85,86]. At a neuropsychological level, executive function impairments (behavioural inhibition, cognitive flexibility, perseveration and monitoring for responses) are associated with RRBs and ED or anxiety; and deficits in the theory of mind (ToM), and alexithymia are also related to difficulties in ED in ASD [25,27,29,78,87,88,89]. Functionally, MS may serve as a self-regulatory coping mechanism to manage physiological arousal activated by anxiety when more adaptive strategies are unavailable [47,75,90], potentially increasing environmental predictability [19,23,24,82,91]. However, MS is likely multifactorial and can also be driven by sensory processing differences or intrinsic pleasure [21,23,25,76,92].

The odds ratio for the LMS–ADS relationship was greater than that for LMS–ED, though this exploratory finding must be viewed in light of the limited ADS sample size. Notably, ADS and ED are highly correlated (rs = 0.736, *p* < 0.001) in our study and difficulties in emotion regulation partly account for the elevated rates of anxiety and depression observed in individuals with ASD [40]. Nevertheless, these constructs are not equivalent, and maintaining a distinction between them is crucial, as the regulation of an emotion is a separate process from the experience of the emotion itself [38]. Children with ASD often face lifelong challenges with both reactive and regulatory components of emotion [27], which may manifest as internalizing (anxiety/depression) or externalizing (aggression/irritability) problems, depending on the moderators of the ED [34,38,39,40,93,94,95].

Specifically, consistent with earlier studies [17,27,32,91,95], age influenced the relationship between LMS and ADS but exerted no impact on the LMS–ED association in the current analysis. Although both ED and MS can persist from toddlerhood into adulthood [27,32,39,42,50,73,74,77], our findings align with the expected developmental shift toward internalizing or complex profiles [17,26,29,52]. Although gender and intellectual disability (ID) were initially considered as potential covariates, they were ultimately excluded from the final multivariable models because preliminary analyses confirmed no significant relationship with our main outcomes. Specifically, ID and gender were not related to ED, ADS, or LMS in our study. This lack of association for ED aligns with previous reports [25,27,28,31,95]. In contrast, while a positive relation of MS with ID is well-documented in the literature [17,96], our findings suggest that significant differences may emerge when ID severity is considered. On the other hand, evidence regarding the relationship between sex and MS remains inconsistent in prior studies [17,96]. The social reciprocity and non-verbal communication index did not modify the LMS-ED and LMS-ADS association, which is not consistent with some reports [17,31,96]. These discrepancies underscore that the interplay between communication abilities, cognitive status, and repetitive behaviours is highly heterogeneous. Future longitudinal studies employing targeted designs are warranted to explicitly examine the influence of gender, ID severity, fine-grained cognitive profiles, and expressive language levels, thereby untangling how age-dependent modifications in behavioural repertoires and emotional regulation capacity interact and how MS–ED and EMS-ADS coupling shifts across distinct neurodevelopmental subgroups.

As clinical implications, should a robust association between MS and ED be further confirmed, these results would emphasize the need for a systematic assessment of LMS frequency and a functional analysis of its underlying mechanisms to guide targeted, evidence-based interventions [97,98,99,100,101,102]. Such analyses should consider multiple variables, including ED, age, level of ID, autism severity, sensory reactivity, and executive functioning, among others [17]. Likewise, indications for intervention on MS should be carefully weighed based on its impact on everyday functioning and social participation [17,36], as well as on the child’s developmental stage, given the regulatory role that they may play in children with ASD who have not yet developed more complex emotional management strategies [35].

While these findings offer valuable preliminary insights to guide clinical development, future prospective studies will be essential to establish causality, directionality, and the specific regulatory role of motor stereotypies; consequently, our clinical implications are framed as hypotheses and directions for future research. Nevertheless, these findings highlight potential targets for intervention, suggesting that future clinical models might benefit from integrating emotional regulation strategies into treatment when LMS are frequent, with a dual focus on reducing reliance on maladaptive emotional regulation strategies and fostering more adaptive coping mechanisms [34]. In this context, evidence-based interventions on ED in ASD children include medication management (aripiprazole, risperidone) [103,104,105,106], cognitive behaviour therapy, behavioural interventions, and parent training [30,32,101]. Naturalistic approaches to promote generalization [28,30] and early family-centred strategies warrant investigation as potential avenues to improve the children’s self-regulatory capacity and family well-being [31,42,107]. Moreover, cognitive-behavioural therapy reduces anxiety, and social skills interventions can decrease anxiety and depressive symptoms in children with ASD [108]. Future longitudinal and interventional studies are required to explicitly establish whether emotional regulation-focused treatments could in turn attenuate MS, shedding light on the dynamic interplay between these behaviours [47,97].

While this study contributes to the sparse literature regarding the association between MS and ED in children with ASD, this study has several limitations. The overall sample size and the prevalence of frequent LMS (32%) may affect the statistical power of the models; while given the small subgroup of participants with clinical ADS (17.2%), its corresponding estimates must be considered exploratory and require replication in larger cohorts The cross-sectional design precludes causal inferences about the association between LMS and ED or ADS. The use of retrospective data also limited the inclusion of more specific measures of MS [15,109,110], although prior work suggests that ADI-R RRB scores are strongly associated with the Repetitive Behavior Scale-Revised (RBS-R) scores [3,15,111]. The reliance on caregiver-rated questionnaires (such as the ADI-R and CBCL) with the lack of direct observational assessments, introduces potential informant-related biases; however, incorporating parent reports is both deliberate and necessary for participants aged 3 to 11 years, because children with ASD frequently experience difficulties with insight and emotional awareness [93]. In addition, the temporal alignment of the measurements could be questioned, as the ADI-R assesses lifetime RRBs while the CBCL-DP reflects current functioning. Nonetheless, because both MS and ED could exhibit long-term persistence in ASD [17,30,31,42,77], it is plausible that their interaction remains longitudinally. Potential mediators or moderators of the MS–ED/MS–ADS relationship, such as sensory processing, cognitive ability, inattention, executive functions, alexithymia, parenting context, and comprehensive data on psychotropic medications, were not systematically examined in this study [23,25,31,41,74,76,92,112,113,114,115,116,117,118]. Although expressive language correlated with ADS was excluded from multivariate models to avoid multicollinearity with the social reciprocity and non-verbal communication index. Finally, the sample was restricted to children under 12 years of age, most with relatively preserved language and cognitive skills, and included few girls; therefore, the generalizability of these findings to older individuals, those with greater impairment, and more sex-balanced samples remains uncertain.

## 5. Conclusions

In conclusion, this study provides empirical evidence that frequent lifetime motor stereotypies in children with ASD are significantly associated with emotional dysregulation and anxiety/depression symptoms, independently of ASD severity. Age modified the relationship between lifetime motor stereotypies and anxiety/depression symptoms. Longitudinal studies are needed to determine the direction and causality of these associations. Due to low prevalence of anxiety/depression symptoms in our sample, results regarding this specific construct must be considered exploratory. Identifying ASD subgroups based on the frequency of motor stereotypies may facilitate the development of targeted diagnostic tools and tailored interventions for emotional regulation, potentially mitigating the risk of future emotional disorders. Future research should evaluate whether interventions aimed at improving emotional regulation can in turn reduce the occurrence of motor stereotypies. Furthermore, differences observed between motor stereotypies and other stereotyped behaviours in relation to ED underscore the importance of distinguishing between low- and high-order RRBs in the clinical evaluation of ASD.

## Figures and Tables

**Table 1:** Sociodemographic and clinical characteristics.

Sample Characteristics:	Total Sample *N* = 151
**Sociodemographic characteristics:**	
Social status *n* (%)	
Upper-upper middle/low middle/working-lower class	24 (17.9%)/87 (64.9%)/23 (17.1%)
Age Median (IQR)	7 (4)
Sex *n* (%) Male/Female	126 (83.4%)/25 (16.6%)
**Clinical characteristics:**	
ID (IQ ≤ 70) *n* (%)	27 (18.8%)
Language delay *n* (%)	56 (39.7%)
Expressive language level *n* (%)	
Preverbal or single words/Simple phrases/Fluent speech	12 (9.2%)/26 (19.8%)/93 (71%)
**ADI-R outcomes**	
**Diagnostic algorithm** Median (IQR)	
A. Qualitative impairments in reciprocal social interaction (Cut-off: 10)	15 (9)
B. Qualitative impairments in communication in verbal subjects (Cut-off: 8)	12 (6)
B. Qualitative impairments in communication in non-verbal subjects (Cut-off:7)	7 (4)
C. Repetitive behaviours and stereotyped patterns (Cut-off: 3)	4 (3)
Encompassing preoccupation or circumscribed pattern of interest (C1)	1 (1)
Apparently compulsive adherences to non-functional routines or rituals (C2)	1 (2)
Stereotyped and repetitive motor mannerisms (C3)	1 (2)
Preoccupations with part-objects or non-functional elements of materials (C4)	1 (1)
D. Development (Cut-off: 1)	3 (2)
**Frequency of LMS *n*** (%) Frequent/occasional/absent	
Stereotyped and repetitive motor mannerisms	49 (32.4%)/35 (23.2%)/67 (44.4%)
Hand and finger mannerisms	25 (16.8%)/16 (10.7%)/108 (72.5%)
Complex mannerisms	20 (13.5%)/32 (21.6%)/96 (64.9%)
**RSMB Mean (SD)**	3 (2)
**CBCL outcomes**	
**CBCL subscales and dimensions** Median (IQR)/Clinical cut-off (%)	
Aggressive behaviour	56 (14)/16 (10.6%)
Anxious/depressed	59 (13)/26 (17.2%)
Attention problems	68 (14)/59 (39.1%)
Withdrawn/depressed	64 (16)/55 (36.4%)
Somatic complaints	53 (8)/6 (4%)
Social problems	63 (12)/31 (20.5%)
Thought problems	64 (13)/40 (26.5%)
Rule-breaking behaviour	57 (13)/14 (9.3%)
Internalizing	61 (15)/71 (47%)
Externalizing	58 (14)/45 (29.8%)
Total	63 (12)/64 (42.4%)
**CBCL-DP** Median (IQR)	185 (29)
SED/DESR/without ED (%)	22 (14.6%)/64 (42.4%)/65 (43%)

ID: intellectual disability; IQ: intelligence quotient; ADI-R: Autism Diagnostic Interview—Revised; LMS: lifetime motor stereotypies; RSMB: Repetitive sensorimotor behaviours; CBCL: Child Behavior Checklist; CBCL-DP: Child Behavior Checklist Dysregulation Profile; SED: Severe emotional dysregulation; DESR: Deficient emotional regulation; ED: emotional dysregulation. Note: Values are expressed as median ± interquartile range (IQR) for continuous variables and as absolute frequencies (*n*) and percentages (%) for categorical variables. Percentages are calculated based on available valid data. Total sample size for specific variables may vary due to missing data as follows: Social status (*n* = 17 missing), ID (*n* = 7 missing), Hand and finger mannerisms (*n* = 2 missing), Complex mannerisms (*n* = 3 missing), CBCL Social problem (*n* = 15 missing), CBCL Thought problems (*n* = 16 missing), CBCL Rule-breaking behaviour (*n* = 14 missing), CBCL Total (*n* = 8 missing).

**Table 2:** Univariate logistic regression of global and types of RRB with ED.

Variable	OR (Exp(B))	95% CI	Wald χ^2^	*p*
ADI-R C	1.036	[0.896, 1.198]	0.231	0.631
ADI-R C1	1.025	[0.745, 1.409]	0.022	0.881
ADI-R C2	1.026	[0.766, 1.375]	0.030	0.863
ADI-R C3	1.436	[0.980, 2.103]	3.453	0.063 *
ADI-R C4	0.915	[0.601, 1.393]	0.171	0.679
RSMB	1.083	[0.899, 1.305]	0.709	0.400

ADI-R: Autism Diagnostic Interview-Revised. C: restricted, repetitive, and stereotyped patterns of behaviour; C1: circumscribed patterns of interest; C2: compulsions and rituals; C3: stereotyped and repetitive motor mannerisms; C4: preoccupation with objects; RSMB: Repetitive Sensorimotor behaviours. * *p* < 0.1.

**Table 3:** Univariate analysis of dependent variables (LMS and covariates) with ED.

Variable	Non-Adjusted OR	95% CI	*p*
LMS	2.268	[1.089, 4.725]	0.029 **
Age	1.155	[0.986, 1.352]	0.074 *
Sex	1.542	[0.652, 3.647]	0.324
ID	0.776	[0.335, 1.797]	0.554
Social reciprocity and non-verbal communication index	0.992	[0.950, 1.035]	0.704
Fluent speech	1.804	[0.875, 3.720]	0.110

LMS: lifetime motor stereotypies; ID: intellectual disability. * *p* < 0.1, ** *p* < 0.05.

**Table 4:** Association between LMS and ED: multivariable logistic regression results.

Variable	OR (Exp(B))	95% CI	Wald χ^2^	*p*
LMS	2.576	[1.184, 5.606]	5.692	0.017 **
Age	1.174	[0.997, 1.383]	3.684	0.055 *
Social reciprocity and non-verbal communication index	0.982	[0.938, 1.028]	0.610	0.435

LMS: lifetime motor stereotypies. * *p* < 0.1, ** *p* < 0.05.

**Table 5:** Univariate analysis of dependent variables (LMS and covariates) with ADS.

Variable	Non-Adjusted OR	95% CI	*p*
LMS	2.676	[1.128, 6.349]	0.025 **
Age	1.430	[1.153, 1.773]	0.001 ***
Sex	1.6375	[0.451, 5.938]	0.453
ID	0.750	[0.235, 2.392]	0.628
Social reciprocity and non-verbal communication index	1.017	[0.962, 1.076]	0.534
Fluent speech	5.441	[1.224, 24.177]	0.025 **

LMS: lifetime motor stereotypies; ADS: Anxiety/Depression symptoms; ID: intellectual disability. ** *p* < 0.05, *** *p* < 0.01.

**Table 6:** Association between LMS and ADS: multivariable logistic regression results.

Variable	OR (Exp(B))	95% CI	Wald χ^2^	*p*
LMS	3.310	[1.266, 8.654]	5.960	0.015 **
Age	1.520	[1.202, 1.922]	12.244	0.001 ***
Social reciprocity and non-verbal communication index	1.026	[0.962, 1.095]	0.619	0.431

LMS: lifetime motor stereotypies. ** *p* < 0.05, *** *p* < 0.01.

## Data Availability

The datasets used and/or analyzed during the current study are available at the corresponding author on reasonable request.

## Supplementary Materials

The following supporting information can be downloaded at: https://www.mdpi.com/article/10.3390/children13101363/s1, Table S1: Preliminary analysis of covariates.

## Abbreviations

The following abbreviations are used in this manuscript:

ASD	Autism spectrum disorder
MS	Motor stereotypies
ED	Emotional dysregulation
ADS	Anxiety/depression symptoms
RRBs	Restricted and repetitive behaviours
CBCL	Child Behaviour Checklist
CBCL-DP	Child Behaviour Dysregulation Profile
ADI-R	Autism Diagnostic Interview-Revised
LMS	Lifetime MS
ID	Intellectual disability
RSMB	Repetitive sensorimotor behaviours
