# Supplementary material for: Association of Motor Stereotypies with Emotional Dysregulation and Anxiety/Depression Symptoms in Children with Autism Spectrum Disorder
Source: Children (Basel). 2026 Oct 8;13(10):1363. doi: 10.3390/children13101363 (PMC13703619; doi:10.3390/children13101363)
Supplement: Supplementary file 1 [file children-13-01363-s001.zip › children-4550510-supplementary.pdf]

Supplementary data

Table S1: Preliminary analysis of covariates

Covariates	LMS		ED		ADS	
	**Statistical test**	**p**	**Statistical test**	**p**	**Statistical test**	**p**
Age	2131.5	0.283	2230	0.041**	920	0.001***
Sex	0.710	0.400	0.980	0.322	0.572	0.571
ID	0.121	0.728	0.352	0.553	0.236	0.785
Social reciprocity and non-verbal communication index	1694.5	0.003***	2672	0.644	1462	0.421
Fluent speech	0.782	0.376	2.58	0.108	6.013	0.014**

Selection of statistical tests based on variable types and parametric assumptions: t de Student / Mann-Whitney; Chi-square / Fisher.

LMS: lifetime motor stereotypies; ED: emotional dysregulation; Anxiety/Depression symptoms; ID: intellectual disability.

**p<0.05, ***p<0.01
